# The highly expressed calcium‐insensitive synaptotagmin‐11 and synaptotagmin‐13 modulate insulin secretion

**DOI:** 10.1111/apha.13857

**Published:** 2022-07-02

**Authors:** Jones K. Ofori, Alexandros Karagiannopoulos, Mohammad Barghouth, Mototsugu Nagao, Markus E. Andersson, Vishal A. Salunkhe, Enming Zhang, Anna Wendt, Lena Eliasson

**Affiliations:** ^1^ Department of Clinical Sciences Malmö, Islet Cell Exocytosis, Lund University Diabetes Centre Lund University Malmö Sweden; ^2^ Islet Pathophysiology, Department of Clinical Sciences Malmö, Lund University Diabetes Centre Lund University Malmö Sweden; ^3^ Department of Endocrinology, Diabetes and Metabolism, Graduate School of Medicine Nippon Medical School Tokyo Japan; ^4^ Department of Physiology, Metabolism Research Unit, Institute of Neuroscience and Physiology, Sahlgrenska Academy University of Gothenburg Gothenburg Sweden

**Keywords:** diabetes, exocytosis, insulin secretion, islet, synaptotagmin, type‐2 diabetes

## Abstract

**Aim:**

SYT11 and SYT13, two calcium‐insensitive synaptotagmins, are downregulated in islets from type 2 diabetic donors, but their function in insulin secretion is unknown. To address this, we investigated the physiological role of these two synaptotagmins in insulin‐secreting cells.

**Methods:**

Correlations between gene expression levels were performed using previously described RNA‐seq data on islets from 188 human donors. SiRNA knockdown was performed in EndoC‐βH1 and INS‐1 832/13 cells. Insulin secretion was measured with ELISA. Patch‐clamp was used for single‐cell electrophysiology. Confocal microscopy was used to determine intracellular localization.

**Results:**

Human islet expression of the transcription factor *PDX1* was positively correlated with *SYT11* (*p* = 2.4e^−10^) and *SYT13* (*p* < 2.2e^−16^). Syt11 and Syt13 both co‐localized with insulin, indicating their localization in insulin granules. Downregulation of Syt11 in INS‐1 832/13 cells (siSYT11) resulted in increased basal and glucose‐induced insulin secretion. Downregulation of Syt13 (siSYT13) decreased insulin secretion induced by glucose and K^+^. Interestingly, the cAMP‐raising agent forskolin was unable to enhance insulin secretion in siSYT13 cells. There was no difference in insulin content, exocytosis, or voltage‐gated Ca^2+^ currents in the two models. Double knockdown of Syt11 and Syt13 (DKD) resembled the results in siSYT13 cells.

**Conclusion:**

SYT11 and SYT13 have similar localization and transcriptional regulation, but they regulate insulin secretion differentially. While downregulation of SYT11 might be a compensatory mechanism in type‐2 diabetes, downregulation of SYT13 reduces the insulin secretory response and overrules the compensatory regulation of SYT11 in a way that could aggravate the disease.

## 1 INTRODUCTION

Insulin is secreted from beta cells organized together with other hormone‐secreting cells in the islets of Langerhans. These islets are spread out all through the pancreas. The main function of the beta cells is to produce and release insulin. Insulin is stored in, and upon stimulation released from, large dense‐core vesicles. Insulin is released when blood glucose levels are elevated (energy supply is high) and functions as a signal for the target tissue to take up and store excess glucose for future needs. Insulin is the only hormone that can lower blood glucose levels in this manner and its release is therefore tightly regulated.^1^ The release of insulin occurs through Ca^2+^‐dependent exocytosis. Elevation of intracellular Ca^2+^ is the result of glucose activation of the stimulus‐secretion coupling pathway.^2^ When glucose is metabolized in the beta cell, intracellular ATP rises at the expense of ADP which leads to the closure of K_ATP_ channels and depolarization of the cell membrane. The depolarization in turn opens voltage‐gated Ca^2+^ channels and the influx of Ca^2+^ triggers exocytosis (extensively reviewed in Ref. [1]). In the beta cells, there are a plethora of exocytotic proteins ensuring that insulin is released at the right levels at the right time. Among these proteins are several synaptotagmins (SYTs).

As of date, there are 17 known human SYT isoforms.^3^ The most studied members of the SYT family, for example, SYT1 and 7, function as Ca^2+^ sensors allowing vesicles to fuse with the plasma membrane when the intracellular Ca^2+^ concentration is elevated. In beta cells, SYT7 and SYT9 are considered the main Ca^2+^ sensors for exocytosis.^2^, ^4^ Intriguingly, several of the known SYTs including SYT4, SYT11, and SYT13 lack functional Ca^2+^ binding sites and hence cannot function as Ca^2+^ sensors for exocytosis.^5^ SYTs such as these are called Ca^2+^‐insensitive SYTs and their role in cell physiology in general, and beta‐cell physiology, in particular, is only starting to emerge.

SYT4 has been reported to inhibit exocytosis in hippocampal neurons^6^ and, along the same lines, it was recently suggested that SYT4 plays a role in the maturation of pancreatic beta cells by modulating their Ca^2+^ sensitivity.^7^ Mice with a global knockout of *Syt11* do not survive after birth.^8^ Overexpression of SYT11 leads to a reduction of dopamine release but the effect is probably not on the exocytotic machinery per se but rather that SYT11 inhibits endocytosis which in turn reduces the replenishment of secretory vesicles.^9^ A similar role for SYT11 in neuronal endocytosis has been reported by other groups as well.^3^ In insulin‐secreting cells information on the role of SYT11 is scarce but one group has reported that SYT11 binds to the RNA‐induced silencing (RISC) complex^10^ indicating a role in microRNA regulation and/or viral defense of the beta cell. SYT13 on the other hand has an unknown role in neurons,^3^ but it has been suggested to play a role in beta‐cell development.^11^ SYT13 has also been implicated in tumor cell growth in some non‐excitable cells.^12^

We have previously reported the reduction of *SYT4*, *SYT11*, and *SYT13* gene expression in islets from type‐2 diabetic (T2D) donors compared to healthy controls.^13^, ^14^ Here we expand on those findings with a special focus on the role of SYT11 and SYT13 in insulin secretion.

## 2 RESULTS

### 2.1 SYT11 and SYT13 correlate positively with PDX1 expression

**FIGURE 1:**
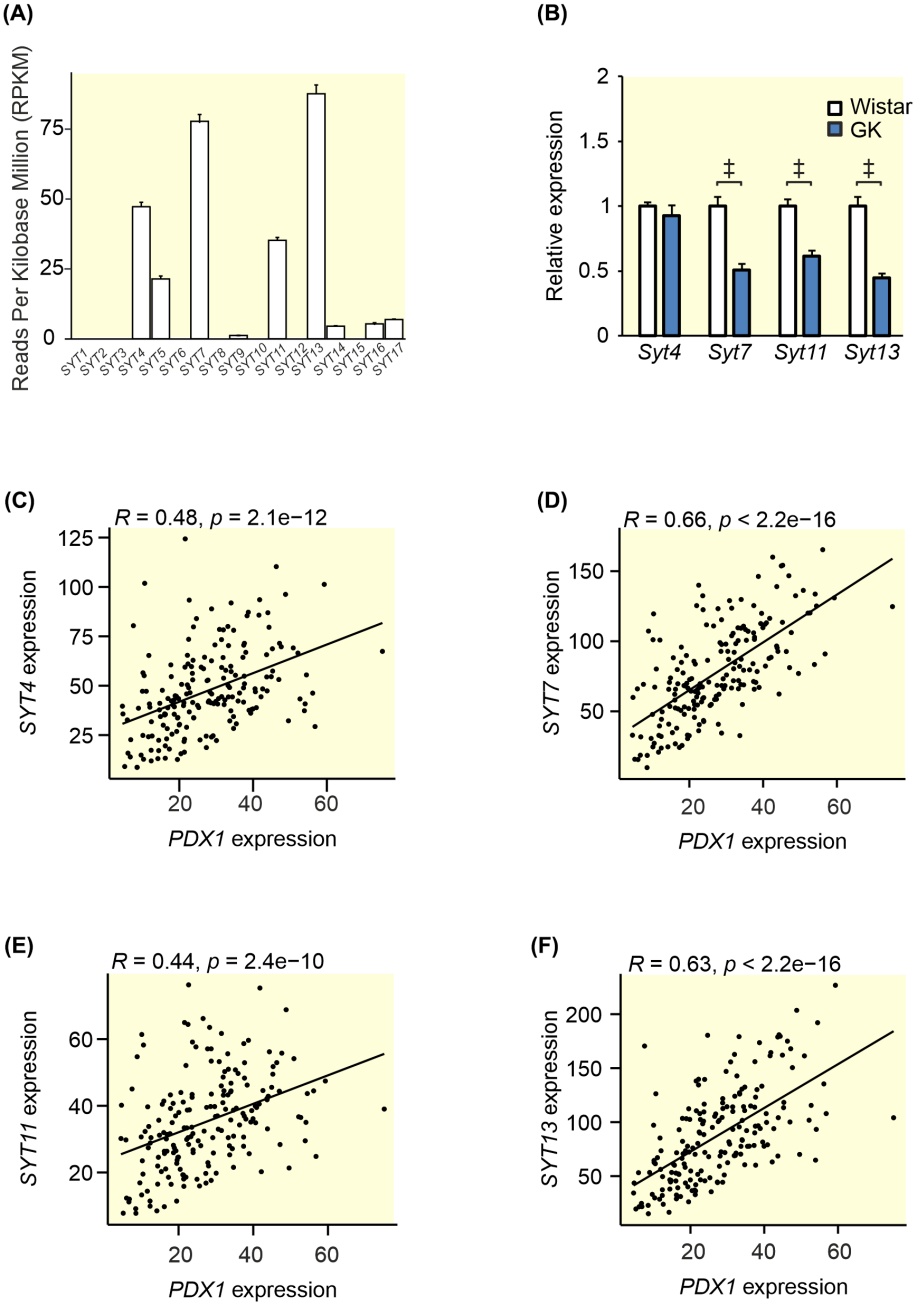
Gene expression of SYTs in human and GK islets and Spearman correlations between the most highly expressed SYTs and PDX1 in human islets. (A) Processed previously described RNA sequencing data^15^ obtained from publicly available repositories (GSE50398, GSE108072) showing *SYT* expression in islets from 188 human donors. Expression is shown as Reads Per Kilobase Million (RPKM). (B) qPCR data from GK rat islets (blue bars) and Wistar control islets (white bars) showing the relative expression of the four most highly expressed SYTs in (A). Spearman correlations between *PDX1* and (C) *SYT4*, (D) *SYT7*, (E) *SYT11*, and (F) *SYT13* using the data in (A) in RPKM. Data are presented as mean ± SEM of 188 donors in (A) and 7–8 rats in (B). *p ≤ 0.05, ^†^ *p* ≤ 0.01, ^‡^ *p* < 0.001.

We have previously shown, with microarray data on islets from 55 donors, that *SYT4*, *SYT7*, *SYT11*, and *SYT13* are the most abundantly expressed SYTs in human islets.^13^ Using previously described RNA‐seq data^15^ obtained from publicly available repositories (GSE50398, GSE108072) on islets from 188 human donors we could confirm these finding (Figure 1A). We have also previously reported that all of these 4 SYTs are downregulated in islets from T2D donors^13^ which led us to investigate if the same is true in Goto Kakizaki (GK) rats, a well‐established T2D model.^16^ As can be seen in Figure 1B; gene expression of *Syt 7*, *Syt 11*, and *Syt 13* is reduced in islets from GK‐rats as compared to Wistar rats, while *Syt 4* remains unchanged.

The question naturally arises why these SYTs are downregulated in islets from T2D donors. It has been reported that the transcription factor PDX1 is downregulated in T2D^17^ and that this transcription factor regulates the expression of *Syt1*.^18^ We, therefore, asked if PDX1 is involved in the regulation of other SYTs. Figure 1C–F shows the Spearman correlation between *PDX1* and *SYT4*, *SYT7*, *SYT11*, and *SYT13* using the same data set as Figure 1A. We found that all of these SYTs show a positive correlation with *PDX1* indicating that they may be under the control of this transcription factor in human islets.

**FIGURE 2:**
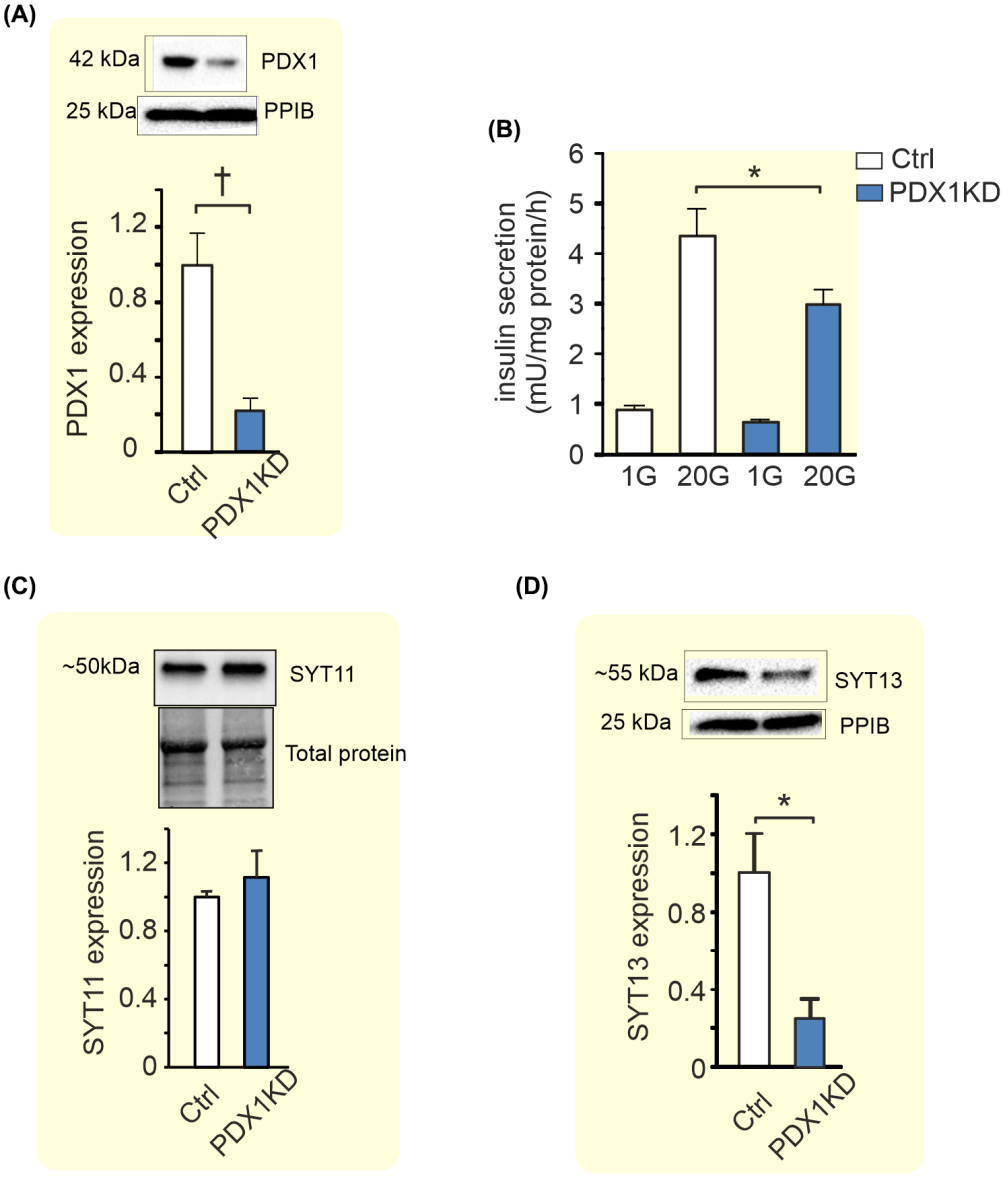
Effects of PDX1 downregulation on insulin secretion, SYT11, and SYT13 protein expression in EndoC‐βH1 cells. (A) Western Blot showing the effects of PDX‐1 downregulation in EndoC‐βH1 on the expression of PDX1. (B) Effect of PDX1 knockdown (blue bars) on insulin secretion at 1 mM glucose (1G) and 20 mM glucose (20G) as indicated in the figure. (C) Western Blot showing the effects of PDX1 knockdown on the protein expression of SYT11 and (D) SYT13. Data are presented as mean ± SEM of four individual experiments in (B–D). *p ≤ 0.05, ^†^ *p* ≤ 0.01.

The role of SYT7 as a calcium sensor of insulin secretion is well described in the literature^5^ and the role of SYT4 as a modulator of calcium sensitivity in the beta cells is also starting to emerge.^7^ However, the role of the two calcium‐insensitive SYTs, SYT11, and SYT13, in mature pancreatic beta cells is still unknown. Therefore, we decided to pursue these two SYTs further. To verify that both SYT11 and SYT13 are regulated by PDX1 we downregulated PDX‐1 in the human beta‐cell line EndoC‐βH1 using siRNA. We achieved a ~80% downregulation of PDX1 in these cells which resulted in decreased glucose‐stimulated insulin secretion (Figure 2A,B) (as previously reported^19^). We could further confirm that downregulation of PDX1 resulted in the reduction of SYT13 at the protein level. However, we did not detect a difference in SYT11 protein with PDX1 downregulation (Figure 2C,D) in the EndoC‐βH1 cell line.

**FIGURE 3:**
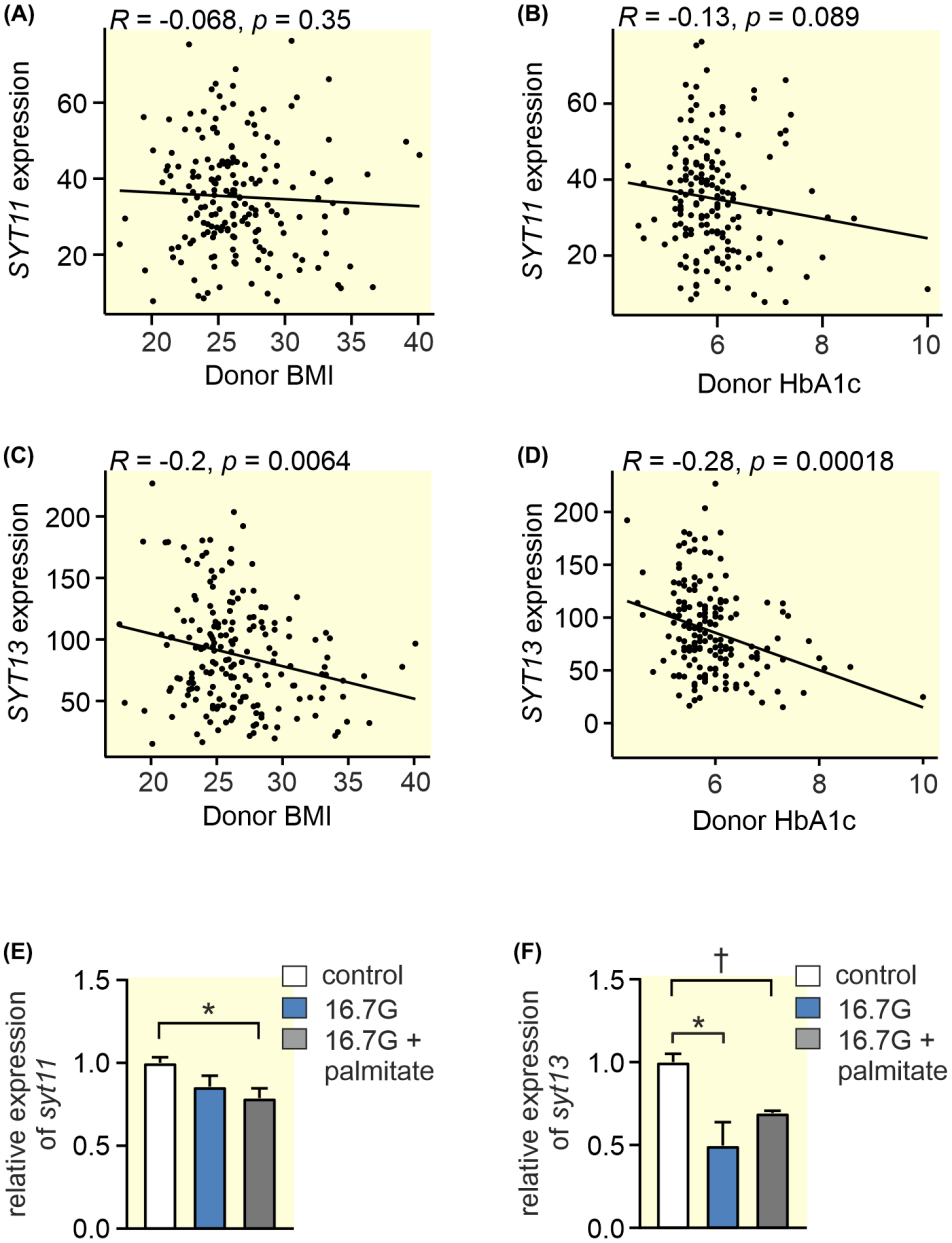
Spearman correlations between *SYT11* or *SYT13* and BMI and HbA1c, and the effect of glucolipotoxicity on their expression. (A) The Spearman correlation using gene expression values as RPKM between (A) *SYT11* and BMI (B) *SYT11* and HbA1c (C) *SYT13* and BMI and (D) *SYT13* and HbA1c in RNA sequencing data obtained from publicly available repositories (GSE50398, GSE108072) from 188 donors.^20^ (E) Relative expression of *syt11* in INS‐1 832/13 cells treated with 16.7 mM glucose (16G; blue bars) or 16.7 mM glucose + 0.5 mM palmitate (16.7G + palmitate; gray bars) compared to control (control; white bars). (F) Relative expression of *syt13* in INS‐1 832/13 cells treated with 16.7 mM glucose (16G; blue bars) or 16.7 mM glucose + 0.5 mM palmitate (16.7G + palmitate; gray bars) compared to control (control; white bars). Data are presented as mean ± SEM of four individual experiments in (E) and (F) *p ≤ 0.05, ^†^ *p* ≤ 0.01.

To further investigate the regulation of SYT11 and SYT13, we determined the correlation between the two SYTs and BMI and HbA1c in the RNA‐seq data from 188 donors.^20^ *SYT13* showed a significant negative correlation with both HbA1c and BMI while *SYT11* showed no correlation with the two traits (Figure 3A–D). Similarly, glucolipotoxic treatment of INS‐1 832/13 cells resulted in a decrease of both *Syt11* and *Syt13* while glucotoxic treatment alone resulted only in the downregulation of *Syt13* (Figure 3E,F).

### 2.2 Downregulation of SYT11 leads to increased basal and glucose‐induced insulin secretion

Next, we investigated the functional role of SYT11 and SYT13, starting with SYT11. In the following experiments we downregulated SYT11 in INS‐1 832/13 cells using siRNA, these cells we call siSYT11 cells while the control cells are called siSCR cells.

**FIGURE 4:**
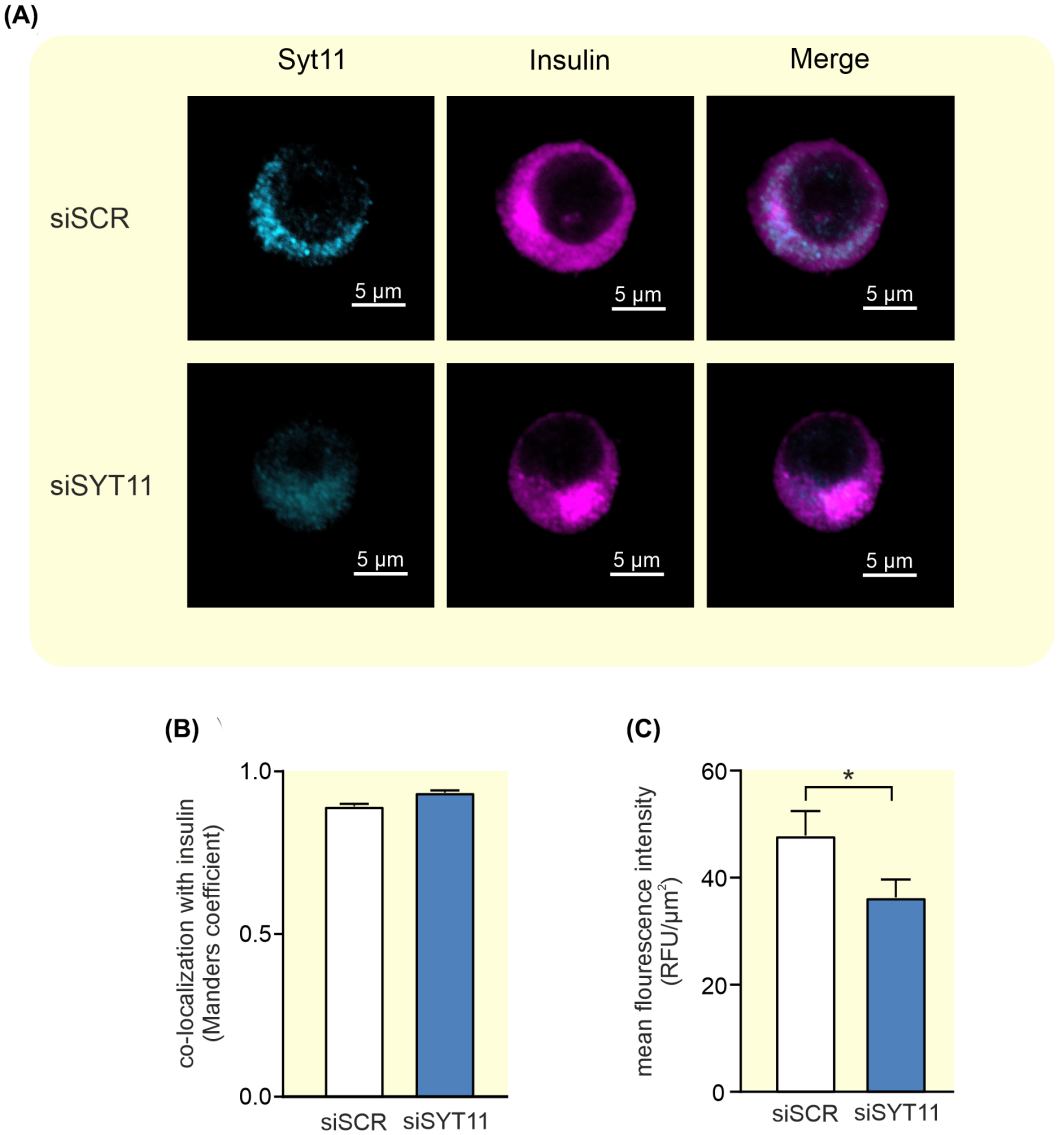
Colocalization between Syt11 and insulin in INS‐1 832/13 cells. (A) Representative confocal images of Syt11 (light blue) and insulin (violet) in INS‐1 832/13 control cells (siSCR) and cells treated with siRNA against *Syt11* (siSYT11). (B) Colocalization of Syt11 and insulin quantified with Manders coefficient in siSCR (white bar) and siSYT11 (blue bar) cells. (C) Quantification of the mean fluorescence intensity of Syt11 in siSCR (white bar) and siSYT11 (blue bar) cells. Data are given as mean ± SEM from 21 to 24 cells. *p ≤ 0.05.

The Ca‐sensitive SYT7 is localized to the insulin‐containing granules, but there is no information on the localization of SYT11. Therefore, we investigated cellular localization of SYT11 using confocal imaging. These experiments indicated a localization to the insulin granules since co‐localization between SYT11 and insulin was high with a Manders coefficient of 0.9 in siSCR cells (Figure 4A,B). We could also confirm the downregulation of SYT11 in siSYT11 cells with a ~30% reduction of the protein immunofluorescence (Figure 4A,C).

**FIGURE 5:**
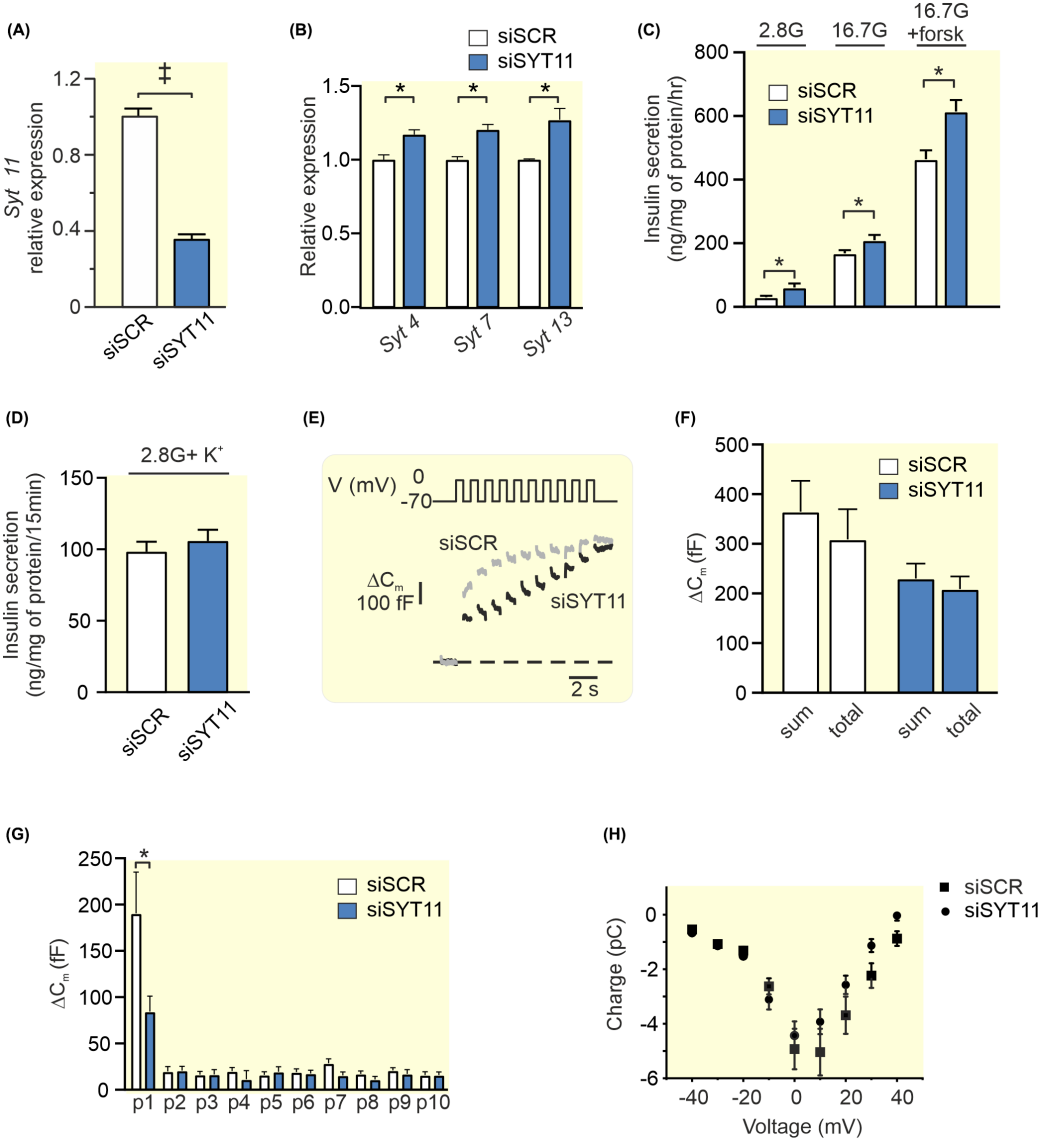
Knockdown of *Syt11* and its effect on insulin secretion and exocytosis in INS‐1 832/13 cells. (A) Expression of *Syt11* after silencing with siRNA (siSYT11; blue bar) relative to its expression using scramble control (siSCR; white bar). (B) Relative expression of *Syt4, Syt7*, and *Syt13* in siSYT11 (blue bars) and siSCR (white bars) cells. (C) Insulin secretion in siSYT11 (blue bars) and siSCR (white bars) cells after stimulation for 1 h in 2.8 mM glucose (2.8G), 16.7 mM glucose (16.7G), or 16.7 mM glucose + 2.5 μM forskolin (16.7G + forsk) as indicated. (D) Insulin secretion in siSYT11 (blue bars) and siSCR (white bars) cells after stimulation with 50 mM K^+^ in 2.8 mM glucose for 15 min. (E) Representative traces of depolarization‐induced increases in membrane capacitance in siSYT11 (black trace) and siSCR (gray trace) cells. (F) Summed capacitance changes (sum) and the total capacitance change (total) for siSyt11 (blue bar) and siSCR (white bar) after a train of ten 500 ms long depolarisations. (G) The capacitance change for siSYT11 (blue bar) and siSCR (white bar) after each individual pulse in (F). (H) Charge–voltage relationships in siSYT11 cells (black circles) compared to their scramble control (black squares). Data are given as mean ± SEM from three to five experiments in (A–D) and 15 cells in (E–G). *p ≤ 0.05, ^†^ *p* ≤ 0.01, ^‡^ *p* < 0.001.

Downregulation of *Syt11* had an efficiency of ~65% on RNA level (Figure 5A) and gave a slight (~20%–30%) but significant compensatory upregulation of *Syt4*, *Syt7*, and *Syt13* expression (Figure 5B).

Insulin secretion at 2.8 mM glucose, 16.7 mM glucose, and in the presence of 16.7 mM glucose + the cAMP raising agent forskolin were all increased in siSYT11 cells (Figure 5C). There was no significant difference in insulin content between the groups (siSCR 1760 ± 390 ng insulin/mg protein vs. siSYT11 2170 ± 580 ng insulin/mg protein; *n* = 4 experiments). We next utilized high K^+^ to investigate depolarization‐induced secretion, but there was no difference in secretory response between the groups (Figure 5D). We decided to further investigate the exocytotic mechanisms in siSYT11 cells using patch‐clamp. Effects of SYT11 downregulation on Ca^2+^‐dependent exocytosis were determined by capacitance measurement (Figure 5E,F). We found that the summed increase in membrane capacitance evoked by ten 500 ms depolarisations (sum) was not significantly different from that of the siSCR cells. Comparing sum to the total capacitance increase between the first and the last pulse in the train (total) gives an estimate of the endocytosis occurring during the train. We found that in neither siSCR nor siSYT11 was there a significant difference between sum and total (365 ± 63 fF vs. 309 ± 61 fF for siSCR and 230 ± 30 fF vs. 209 + 25 fF for siSYT11 cells). When comparing the exocytotic response to each pulse in the train we discovered a surprising decrease in the response to the first pulse in siSYT11 cells compared to control (Figure 5G). A closer examination of the patch‐clamp data revealed endocytosis in 13 out of 15 siSCR cells. In these 13 cells, the average endocytosis amounted to 67 ± 12 fF per cell, while siSYT11cells exhibited endocytosis in 9 out of 15 cells and in those 9 cells endocytosis averaged at 65± 17 fF per cell. We finally asserted that there was no effect on the voltage‐dependent influx of Ca^2+^ with the siRNA treatment and as expected there was no difference in charge (Q) flowing through the channels at physiologically relevant membrane potentials (Figure 5H). There was no significant difference in cell size between siSYT11 and siSCR cells in the electrophysiological experiments (5.74 ± 0.39 pF vs. 5.78 ± 0.38 pF; *n* = 9–13).

### 2.3 Downregulation of SYT13 reduces glucose‐induced insulin secretion

**FIGURE 6:**
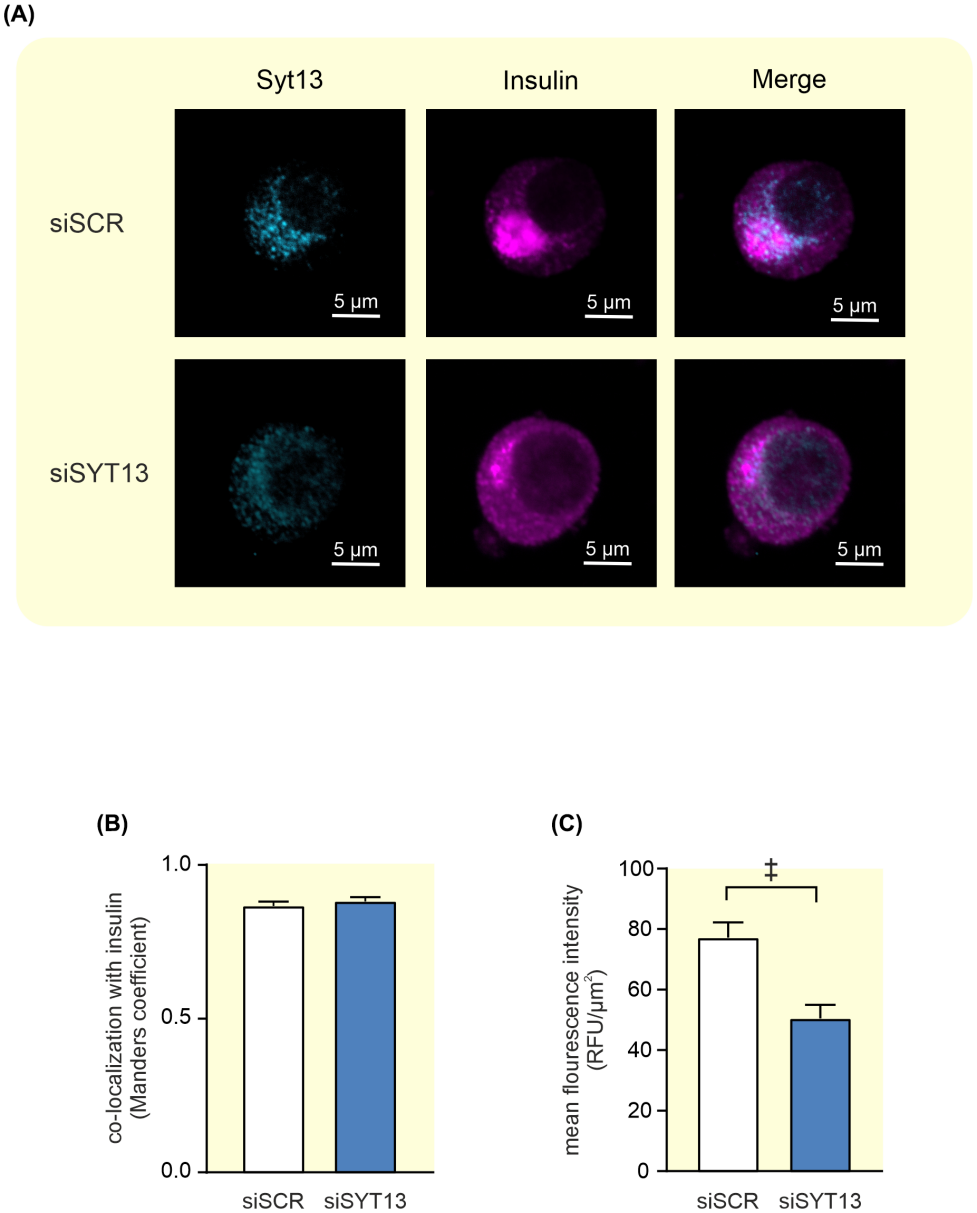
Colocalization between Syt13 and insulin in INS‐1 832/13 cells. (A) Representative confocal images of Syt13 (light blue) and insulin (violet) in INS‐1 832/13 control cells (siSCR) and cells treated with siRNA against *Syt13* (siSYT13). (B) Colocalization of Syt13 and insulin quantified with Manders coefficient in siSCR (white bar) and siSYT13 (blue bar) cells. (C) Quantification of the mean fluorescence intensity of Syt13 in siSCR (white bar) and siSYT13 (blue bar) cells. Data are given as mean ± SEM from 22 to 23 cells; ^‡^ *p* < 0.001.

We thereafter used siRNA to downregulate SYT13 in INS‐1 832/13 cells, these cells we call siSYT13 cells and compared the results with control (siSCR) cells. As with SYT11, SYT13 was found to localize to the large dense‐core vesicles (Figure 6A,B).

**FIGURE 7:**
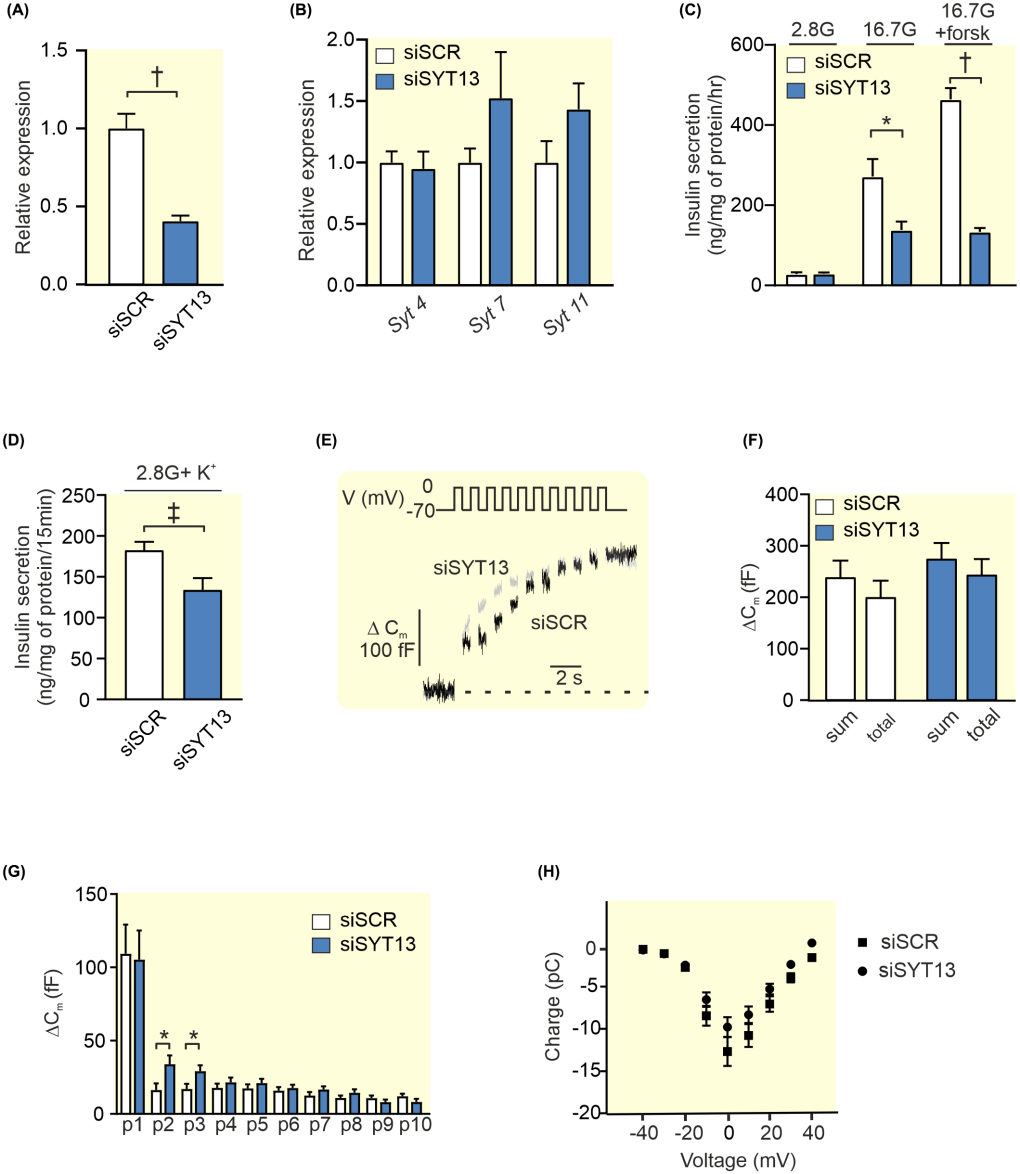
Knockdown of *Syt13* and its effect on insulin secretion and exocytosis in INS‐1 832/13 cells. (A) Expression of *Syt13* after silencing with siRNA (siSYT13; blue bar) relative to its expression using scramble control (siSCR; white bar). (B) Relative expression of *Syt4, Syt7*, and *Syt11* in siSYT13 (blue bars) and siSCR (white bars) cells (C) Insulin secretion in siSYT13 (blue bars) and siSCR (white bars) cells after stimulation for 1 h in 2.8 mM glucose (2.8G), 16.7 mM glucose (16.7G), or 16.7 mM glucose + 2.5 μM forskolin (16.7G + forsk) as indicated. (D) Insulin secretion in siSYT11 (blue bars) and siSCR (white bars) cells after stimulation with 50 mM K^+^ in 2.8 mM glucose for 15 min. (E) Representative traces of depolarization‐induced increases in membrane capacitance in siSYT13 (black trace) and siSCR (gray trace) cells. (F) Summed capacitance changes (sum) and the total capacitance change (total) for siSYT13 (blue bar) and siSCR (white bar) after a train of ten 500 ms long depolarisations. (G) The capacitance change for siSYT13 (blue bar) and siSCR (white bar) after each individual pulse in (F). (H) Charge—voltage relationships siSYT13 cells (black circles) compared to scramble controls (black squares). Data are given as mean ± SEM from three to five experiments in (A–D) and 28–29 cells in (E–H). *p ≤ 0.05, ^†^ *p* ≤ 0.01, ^‡^ *p* < 0.001.

The downregulation of SYT13 on the protein level was confirmed by a ~45% reduction of the protein immunofluorescence in siSYT13 cells (Figure 6A,C). Downregulation of *Syt13* had an efficiency of ~60% on RNA level and we observed no significant compensatory regulation on mRNA level of *Syt4*, *Syt7*, or *Syt11* expression in siSYT13 cells (Figure 7A,B).

We confirmed our previously reported findings^13^ that insulin secretion at 16.7 mM glucose is significantly reduced in siSYT13 cells, hereby ~50%, compared to siSCR cells. We extended these findings by also examining forskolin‐stimulated insulin secretion at 16.7 mM glucose where we found that forskolin could not enhance GSIS in siSYT13cells. (Figure 7C). We also measured K^+^‐induced insulin secretion and found that this was significantly decreased in siSYT13 cells (Figure 7D). Downregulation of Syt13 did not affect insulin content. (siSCR cells 1570 ± 360 ng insulin/mg protein vs. siSYT13 cells 1500 ± 470 ng insulin/mg protein; *n* = 5 experiments).

When we investigated the exocytotic mechanisms using patch‐clamp, we found that there was no difference in exocytosis between siSYT13 and siSCR cells in the summed increase or total increase (sum; total; Figure 7E,F). When analyzing each individual pulse in the train we observed a surprising increase in exocytosis in the 2nd and 3^d^ pulse in siSYT13 cells (Figure 7G). Regarding endocytosis, we noticed that in neither siSCR nor siSYT13 cells were there a significant difference between sum and total(249 ± 33 fF vs 207 ± 33 fF for siSCR and 284 ± 31 fF vs. 254 + 30 fF for siSYT13 cells). Endocytosis occurred in 20 out of 29 siSCR cells. In those 20 cells, endocytosis averaged at 68 ± 14 fF, while siSYT13 cells exhibited endocytosis in 18 out of 28 cells with an average of 64± 14 fF in the endocytotic cells.

As expected, there was no difference between the groups in the voltage‐dependent influx of Ca^2+^ measured as charge (Q) flowing through the voltage‐gated Ca^2+^ channels (Figure 7H) nor was there a significant difference in cell size between siSCR and siSYT13 cells (5.77 ± 0.17 pF vs 6.43 ± 0.35 pF; *n* = 33–37 cells).

### 2.4 Upregulation of exocytotic genes in siSYT11 and siSYT13cells

**FIGURE 8:**
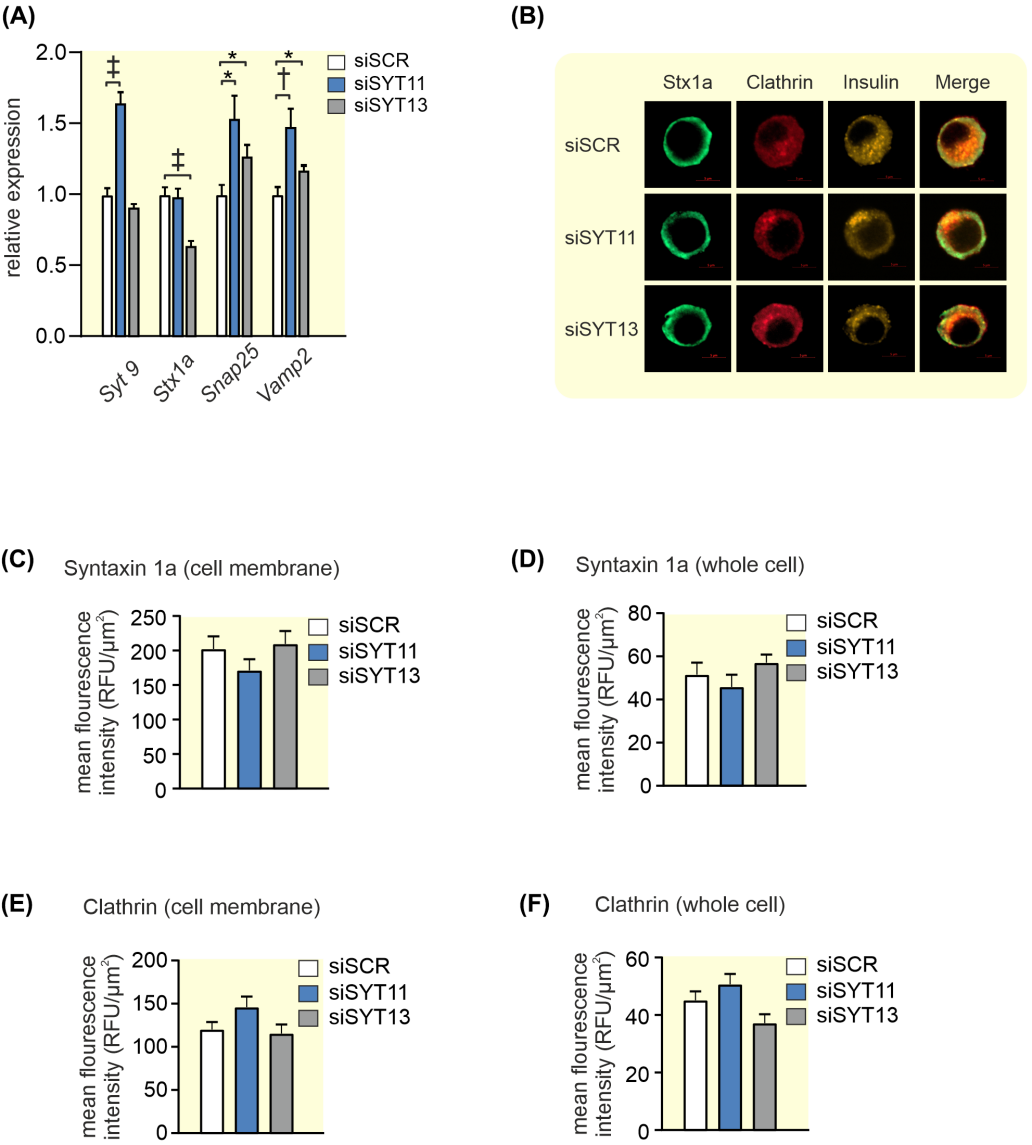
Expression of exo‐ and endocytotic genes in siSYT11 and siSYT13. (A) Relative expression of selected exocytotic genes in siSYT11 (blue bars) and siSYT13 (gray bars) compared to control (siSCR; white bars). (B) Representative confocal images of Syntaxin 1a (Stx1a;green), Clathrin (red), and Insulin (yellow) in a INS‐1 832/13 control cells (siSCR) a siSYT11 cell and a siSYT13 cell. Amount of syntaxin 1a estimated by mean fluorescence intensity in (C) the cell membrane and (D) the whole cell. Amount of clathrin estimated by mean fluorescence intensity in (C) the cell membrane and (D) the whole cell. Data are given as mean ± SEM from four experiments in (A) and 22–26 cells in (C–F). *p ≤ 0.05, ^†^ *p* ≤ 0.01, ^‡^ *p* < 0.001.

In order to better understand the phenotypes in siSYT11 and siSYT13 cells, we investigated the gene expression of selected exocytotic genes in the two systems. We found that in siSYT11 cells *Syt9*, *Snap 25*, and *Vamp2* are upregulated while in siSYT13 cells *Snap 25* and *Vamp2* are upregulated while syntaxin 1a (*Stx1a*) appeared to be downregulated (Figure 8A). However, a further investigation of Stx1a on protein level using immuno‐fluorescence did not confirm a downregulation on protein level (Figure 8B–D). We also investigated the endocytotic protein clathrin but found no difference in either expression or localization of the protein in siSYT11 or siSYT13 cells (Figure 8B,E,F).

### 2.5 Downregulation of both SYT11 and SYT13 reduces glucose‐induced and depolarization‐induced insulin secretion

**FIGURE 9:**
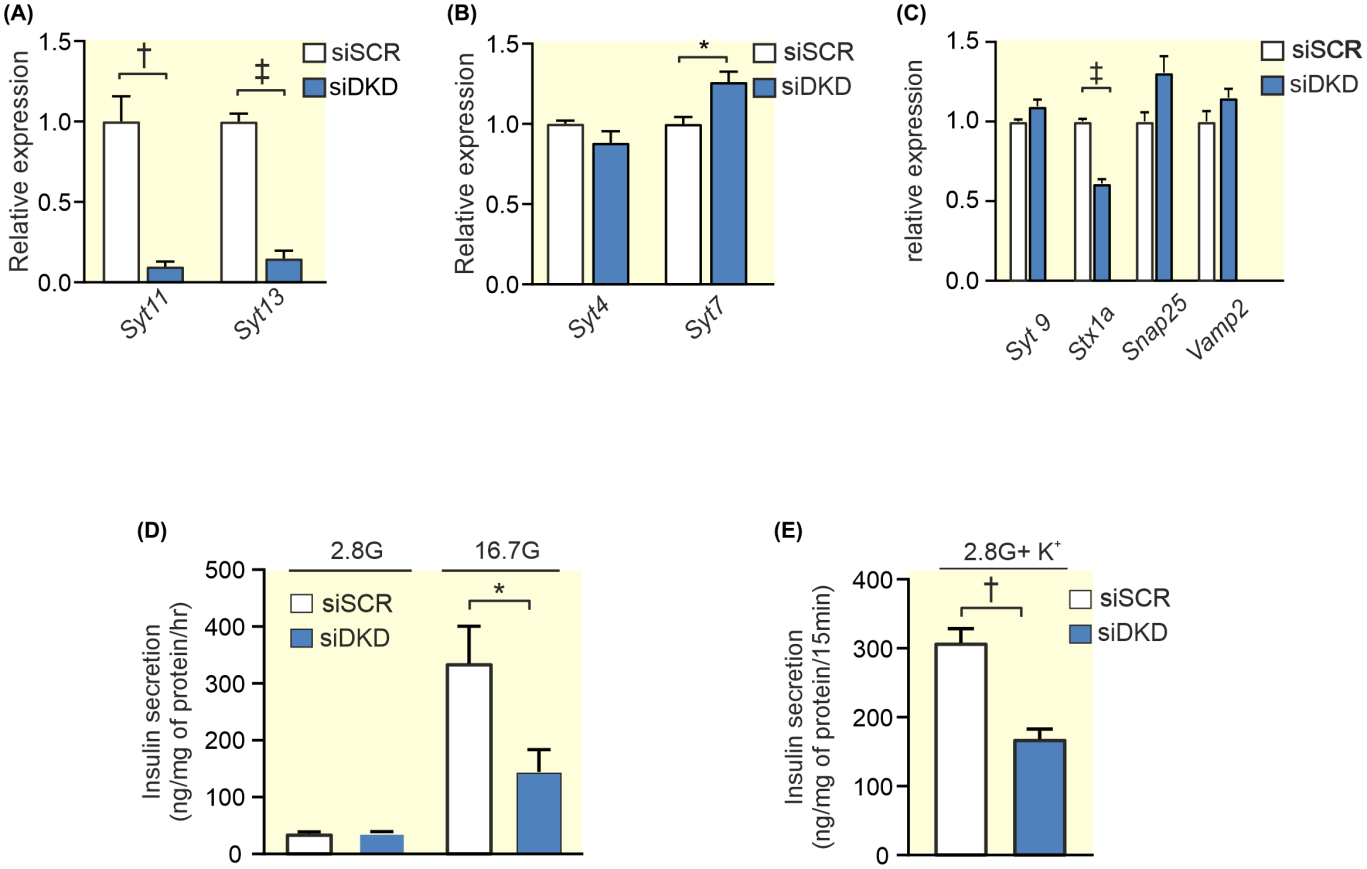
Double knockdown of *Syt11* and *Syt13* and its effect on insulin secretion in INS‐1 832/13 cells. (A) Expression of *Syt11* and *Syt13* (blue bar) after double silencing with siRNA (siDKD) relative to its expression using scramble control (siSCR; white bar). (B) Relative expression of *Syt4* and *Syt7* in siDKD (blue bars) and siSCR (white bars) cells. (C) Relative expression of selected exocytotic genes in siDKD (blue bars) compared to control (siSCR; white bars). (D) Insulin secretion in siDKD (blue bars) and siSCR (white bars) cells after stimulation for 1 h in 2.8 mM or 16.7 mM glucose as indicated. (E) Insulin secretion in siDKD (blue bars) and siSCR (white bars) cells after stimulation with 50 mM K^+^ in 2.8 mM glucose for 15 min. Data are given as mean ± SEM from four experiments. *p ≤ 0.05, ^†^ *p* ≤ 0.01, ^‡^ *p* < 0.001.

Finally, we downregulated Syt11 and Syt13 together in INS‐1 832/13 cells, we call these cells siDKD cells. The cells were then compared with control (siSCR). In the siDKD cells, *Syt11* and *Syt13* were downregulated by 85% and 90%, respectively, which resulted in a slight compensatory upregulation of *Syt7* and a downregulation of *Stx1a* (Figure 9A–C). We found that glucose‐induced insulin secretion in siDKD cells was significantly reduced by ~55% with an unchanged basal insulin secretion (Figure 9D). Similarly, depolarization‐induced insulin secretion was down by ~45% in these cells (Figure 9E) and as expected insulin content also remained the same (siSCR cells 1430 ± 220 ng insulin/mg protein vs. siDKD cells 1290 ± 260 ng insulin/mg protein; *n* = 4 experiments).

## 3 DISCUSSION

We have shown that the two calcium‐insensitive SYTs; SYT11 and SYT13 play a role in shaping insulin secretion. We find no evidence that this is due to effects on endocytosis or exocytosis although downregulation of the two SYTs does affect gene expression of some other exocytotic proteins.

The role of the calcium‐insensitive SYTs has for long remained an enigma. Out of the 17 known human SYT isoforms, eight do not bind calcium.^3^ Thus, clearly there must be other roles for this versatile family of proteins than merely being the calcium sensors of exocytosis. Human islets express several SYTs. Some are barely detectible (e.g., *SYT2, 3, 6, 10, 12*, and *15*; Figure 1A) while others are more highly expressed. It is interesting that out of the four most highly expressed SYTs in human islets only one (SYT7) is calcium‐sensitive. The other three (SYT4, 11, and 13) are not. In order to understand the role of individual SYTs, it is helpful to know in which cell types they are expressed. In sorted human alpha and beta cells, *SYT 3, 4, 13, 15*, and *16* all have a nominal *p*‐value < 0.05 for adult beta vs alpha cells.^21^ Using a different approach Segerstolpe et al. profiled single‐cell transcriptomes from islets cells and they found that *SYT13* and *SYT7* were differentially expressed with a higher mean expression in beta cells, while *SYT5* was differentially expressed with a higher mean expression in alpha cells.^22^ We have previously published that all top four islet expressed SYTs (SYT4, 7, 11, 13) show a negative correlation with HbA1c.^13^ Here we have used a larger cohort and found that while SYT13 negatively correlates with both HbA1c and BMI, SYT11 does not. The question is if the lower expression of these SYTs is a cause or a consequence of the diabetic state. When subjected to glucotoxic or glucolipotoxic conditions *Syt13* is downregulated as a consequence of both treatments while both gluco‐ and lipotoxicity are necessary to downregulate *Syt11* in INS‐1 832/13 cells. Thus, although *Syt11* and *Syt13* are similarly regulated the mechanisms do not seem to be exactly the same. The fact that the transcription factor PDX1 positively correlates with the expression of all top four SYTs (see Figure 2) suggests that the lower expression of these SYTs might be partly explained by the lower PDX1 levels in islets from T2D donors.^17^ PDX1 is a transcription factor that plays a key role in pancreatic development and beta‐cell function and is known to regulate a number of key beta cell genes including the insulin gene. Moreover, PDX1 is known to be downregulated in T2D probably due to the hyperglycemic state.^17^ Only SYT13 and not SYT11 protein levels were reduced with downregulation of PDX1 in EndoC‐βH1 cells. If the latter is due to differential regulation of SYT11 in EndoC‐βH1 cells compared to human islets is unclear. Nevertheless, it seems likely that as blood glucose increases in the diabetic state, PDX1 levels are reduced which in turn leads to a downregulation of SYT13 and possibly several other SYTs.

Since T2D islets have reduced expression of *SYT11* and *SYT13* ^13^ it is important to understand their impact on insulin secretion. Our data indicate that reduced expression of *SYT11* leads to increased insulin secretion. However, depolarization‐induced (K^+^) insulin secretion, as well as exocytosis, is unchanged. Insulin secretion is controlled by a triggering and an amplifying pathway, where the amplifying pathway is fuelled by glucose‐derived metabolites.^23^ Mathematical modeling further suggests that the amplifying pathway enhances mobilization and priming of the insulin granules.^24^ It is possible that SYT11 has an effect on the amplifying pathway of insulin secretion. The observed colocalization of Syt111 with insulin (suggesting that SYT11 resides on the insulin granules) supports this possibility. We have not investigated if there is a shift in glucose dependence in these cells but data on mouse islets indicate that the continuous depolarization triggered by high K^+^ and the peak amplitude of the membrane oscillations triggered by high glucose reach approximately the same value.^25^ We hypothesize that the reduced expression of *SYT11* in T2D attempts to compensate for the reduced insulin secretion that occurs with the disease. In siSYT11 cells several exocytotic proteins are upregulated, which might contribute to the increased insulin secretion. It is unclear if this is a direct or compensational effect of Syt11 downregulation.

In neurons, SYT11 has been reported to play a role in endocytosis and therefore we investigated if this is true in siSYT11 cells.^9^ In primary rodent beta cells endocytosis has been described as a slower process than exocytosis.^26^ This is however not as clearly the case in the cell line INS‐1 832/13 illustrated by the fact that we can measure endocytosis in the majority of both the siSCR and siSYT11 cells. Although we find no difference in the total endocytosis between siSYT11 and control or in the expression and localization of clathrin there are slightly fewer cells exhibiting endocytosis in siSYT11. We can therefore not fully rule out that SYT11 plays a role in endocytosis also in beta cells.

Another suggested possibility is that SYT11 takes part in microRNA regulation of protein levels in the cell.^10^ Interestingly, we have previously shown that SYT11 is a target for microRNAs with altered expression due to glycemic status in human islets, while SYT13 is not.^27^ It is well known that insulin secretion is tightly regulated by several mechanisms including microRNA regulation and that failure in this regulation could lead to T2D (reviewed in Ref. [28]). The role of SYT11 in such a regulation is clearly intriguing and merits further investigation.

The immune fluorescence data suggest that also Syt13 resides on the insulin granules. Downregulation of Syt13 leads to a decrease in glucose‐stimulated insulin secretion and mimics the situation in T2D patients. The fact that there is a decrease also in K^+^‐induced insulin secretion suggests defects in the first phase of insulin secretion. Interestingly inducing higher cAMP levels using forskolin does not amplify insulin secretion in siSYT13 cells, indicating that Syt13 is involved in the cAMP‐regulated amplification of insulin secretion. cAMP has been suggested to enhance the readily releasable pool of insulin vesicles and the recruitment of new vesicles as well as sensitize the exocytotic machinery to Ca^2+^, thus cAMP affects both the first and the second phase of insulin secretion.^29^ To our surprise we could not detect a difference in exocytosis measured by capacitance measurements between siSYT13 and siSCR cells. The reason for this is unclear but one possibility is the difference in depolarization strength between the two experiments. In the exocytosis measurements, we depolarize the cells to 0 mV while the membrane potential with high K^+^ or the highest amplitudes in the membrane oscillations induced by 16.7 mM glucose are reported to reach a maximum of ‐10 mV in mouse islets^25^ and probably lower in INS‐1 cells.^30^ It is, therefore, possible that a shift in the glucose‐response curve of insulin secretion in siSYT13 cells could partly explain this finding.^31^

To mimic the situation in T2D we downregulated Syt11 and Syt13 at the same time (DKD). The phenotype of these cells closely mimicked the siSYT13 cells, indicating that downregulation of Syt13 overrules the compensatory effects of downregulated Syt11.

One might argue that the findings presented here are contaminated by the effects of compensatory upregulation of other exocytotic genes including other SYTs. Based on previous publications^2^, ^7^ and the findings in this publication, we cannot completely rule out interference in the case of siSYT11. However, in the case of siSYT13 or in the siDKD cells if anything upregulation of the other exocytotic genes would drive insulin secretion in the opposite direction (as would be expected from a compensatory upregulation).

In conclusion, this study highlights the fact that although many of the highly expressed SYTs in pancreatic beta cells lack the ability to sense calcium, they still bring important contributions to insulin secretion. We show that downregulation of SYT11 and SYT13 in beta cells, both of which occur in T2D, affects insulin secretion differently with siSYT13 being dominant. Our data present possible mechanisms involved, but the field certainly warrants further attention since normalization of the beta‐cell function is key to battling diabetes.

## 4 MATERIALS AND METHODS

### 4.1 Islets and cell culture

Goto‐Kakizaki (GK/LU colony, bread in‐house)^16^ and Wistar control rats (Taconic) age 11–14 weeks were sacrificed in a CO_2_ chamber and the islets were isolated using collagenase digestion as previously described.^25^ All animal procedures were approved by the Malmö/Lund Committee for Animal Experiment Ethics, Sweden.

INS‐1 832/13 cells^32^ were maintained in RPMI 1640 medium (HyClone, UT, USA) containing 11.1 mM D‐glucose (HyClone, UT, USA), supplemented with 10% (v/v) heat inactivated fetal bovine serum (FBS), 100 IU/ml of penicillin (HyClone, UT, USA), 100 μg/ml of streptomycin (HyClone, UT, USA), 10 mM HEPES (HyClone, UT, USA), 2 mM L‐glutamine (HyClone, UT, USA), 1 mM sodium pyruvate (HyClone, UT, USA), and 50 μM 2‐mercaptoethanol. Glucolipotoxic conditions were induced by incubating the cells in 16.7 mM glucose and 0.5 mM palmitate for 48 h.

EndoC‐βH1 cells (EndoCells, Paris, France)^33^ were seeded in matrigel/fibronectin‐coated (100 μg/ml and 2 μg/ml, respectively, Sigma‐Aldrich) culture vessels in DMEM containing 5.6 mmol/L glucose, 2% BSA fraction V, 10 mmol/L nicotinamide, 50 μmol/L 2‐mercaptoethanol, 5.5 μg/ml transferrin, 6.7 ng/ml sodium selenite, 100 U/ml penicillin, and 100 μg/ml streptomycin.

All cells were incubated in a humidified atmosphere with 5% CO_2_ at 37°C.

### 4.2 Transfection

One day prior to transfection ~400.000 of INS‐1 832/13 cells/well were seeded in antibiotic‐free RPMI 1640 media in a 24‐well‐plate. For downregulation of Syt11; *Silencer® Select Pre‐Designed siRNA* against Syt11 (50 nM; s133472; #4390771; Life Technologies CA, USA) was used and *Silencer*® Select Negative Control No. 2 siRNA was used as negative control (50 nM; #4390846; Life Technologies CA, USA). Transfection was performed according to the manufacturer's protocol using lipofectamine® RNAiMAX Reagent (Invitrogen, CA, USA). The cells were transfected 72 h prior to experiments. For downregulation of SYT13, a similar protocol as above was used: a final transfection volume of 600 μl per well contained 25 nM of *Silencer® Select Pre‐Designed siRNA* against SYT13 (s135284; #4390771) or Silencer® Select Negative Control No. 2 siRNA in Opti‐MEM reduced serum media and 1.5 μl of Lipofectamine RNAiMAX. A second transfection was performed 24 h after the first transfection. For co‐transfection of Syt11 and Syt13 (siDKD), the final transfection volume of 600 μl per well contained 25 nM Syt13 (s135284; #4390771) and 25 nM of Syt11 (s133472; #4390771) or 50 nM Silencer® Select Negative Control No. 2 siRNA (#4390846).

For transfection in EndoC‐βH1 cells, 180.000 cells per well were seeded in a 48‐well plate containing 150 μl antibiotic‐free medium 1 day before transfection. The following day, the cells were transfected with *Silencer® Select Pre‐Designed* siPDX1 (#4392420) using Lipofectamine RNAiMAX (Thermo Fisher). A final transfection volume of 200 μl per well contained 50 nM of the siRNA in Opti‐MEM reduced serum media and 0.5 ml of Lipofectamine RNAiMAX (Life Technologies, SanFrancisco, CA, USA). A second transfection was performed 24 h after the first transfection. All functional experiments were performed 72 h after the first transfection.

### 4.3 Immunostaining

INS‐1 832/13 cells were cultured in μ‐Slide (chambered coverslip) with 8 wells (LAB‐TEK, 154534) 1 day prior to immunostaining. The cells were first washed twice with PBS (Hyclone) and fixed with 3% PFA‐K‐PIPES and 3% PFA‐Na2BO4 for 5 and 10 min, respectively, followed by permeabilization with 0.1% Triton‐X 100 for 30 min at room temperature. The cells were incubated with the blocking solution containing 5% normal donkey serum (Jackson immunoresearch) in PBS for 30 min. Primary antibodies against Syt11 (1:50, # E‐AB‐10622, Elabscience), Syt13 (1:100, # ab‐154 695, Abcam), insulin (1:400, #16049, Progen), Syntaxin1a (1:100, #VAP‐SV064‐E, ENZO), or Clathrin (1:100, #MA1‐065, Invitrogen) were diluted in blocking solution and incubated overnight at 4°C. Immunoreactivity was quantified using fluorescently labeled secondary antibodies either Alexa Fluor 488, donkey anti‐rabbit (1:300, Jackson ImmunoResearch), or Cy 3, donkey anti‐guinea pig (1:300, Jackson ImmunoResearch). The Confocal images were acquired using a Zeiss LSM 800 on a 63× oil immersion objective. The fluorescent intensity and Manders colocalization coefficient were analyzed with software ZEN (2.6 Blue edition).

### 4.4 Insulin secretion assay

For insulin secretion assay, INS‐1 832/13 cells were plated in triplicate for each condition and the assay was performed as previously described.^34^ Stimulation of insulin secretion was performed with 2.8 mM glucose, 16.7 mM glucose, 16.7 mM glucose + 2.5 μM forskolin, or 2.8 mM glucose + 50 mM K^+^. In the latter case NaCl_2_ was substituted with KCl in equimolar ratio. Prior to insulin secretion in EndoC‐βH1 cells, the cells were transferred to a complete glucose starvation medium containing 2.8 mM for 18 h and insulin secretion assay was performed as previously reported.^35^

Secreted insulin levels were measured using Mercodia High Range Rat Insulin ELISA (# 10‐1145‐01), Mercodia human Insulin ELISA (#10‐1113‐0) or Coat‐a‐Count RIA (Millipore Corporation, MA, USA). Insulin secretion measurements were normalized to total protein content from the same well.

### 4.5 Protein extraction

Prior to protein extraction, the INS‐1 832/13 cells were washed in ice‐cold PBS and incubated on ice for ~15 min in RIPA buffer (Radio Immunoprecipitation Assay); 150 nM NaCl, 1% TritonX‐100, 0.1% SDS, 50 mM Tris‐Cl, pH 8 and EDTA‐free protease inhibitor (Roche, NJ, USA). The resulting cell lysate was harvested, transferred to pre‐cooled tubes, and centrifuged at 4°C for 15 min at 14.000 *g*. The supernatant was carefully transferred to new tubes and stored at −20°C. Protein content of the supernatant was analyzed using the BCA assay (Pierce®BCA Protein Assay Kit #23227, IL, USA) and BioRad Model 6870 Microplate Reader. The insulin content in the samples was determined using Mercodia High Range Rat Insulin ELISA (# 10‐1145‐01), Coat‐a‐Count RIA (Millipore Corporation, MA, USA), or Mercodia human Insulin ELISA (#10‐1113‐0).

### 4.6 RNA Extraction, RT PCR, and qPCR

INS‐1 832/13 cells or GK/Wistar rat islets (75‐200 islets) were washed in ice‐cold PBS, lysed in Qiazol (Qiagen, MD, USA), loosened by pipetting, and transferred to microfuge tubes. The cells were homogenized by vortexing the microfuge tubes for 1 min and stored at −20°C until RNA extraction. MiRNeasy®Mini Kit protocol (#217004, Qiagen, Germany) was used for total RNA extraction. RNA concentration was measured using NanoDrop (ND‐1000 Spectrophotometer).

RT‐PCR was performed on the PTC‐200 Peltier Thermal Cycler. For mRNA according to the protocol of High Capacity cDNA Reverse Transcriptase Kit (#4368814, Applied Biosystems, CA, USA) using Random Primers. qPCR was performed in triplicates in a 384‐well plate using Applied Biosystems QuantStudio (TM) 7 Flex RT‐PCR system under default cycling parameters. Specific primers and probes from TaqMan®Gene Expression Assays (Applied Biosystems, CA, USA): *syt4* (Rn01157571_m1), *syt7* (Rn00572234_M1), *syt11* (Rn00581475_m1),*syt13* (Rn00578161_m1), *Syt9* (Rn00584114_m1), *Snap25* (Rn00578534_m1), and *Vamp2* (Rn00360268_g1) were used to measure mRNA levels. The endogenous control assays used for the mRNAs were *hprt1* (Rn01527840_m1) and *ppia* (Rn00690933_m1). Relative expressions were calculated using the ΔΔCt method.

### 4.7 Western blot

Total protein, 15 μg/ml was extracted at 72 h post‐transfection from EndoC‐βH1 and separated by 4%–15% TGX Stain‐Free gels (Bio‐Rad, Hercules, CA, USA). The gels were then activated with UV light for 1 min to visualize total protein on the blotted LF PVDF membrane (Bio‐Rad). Protein was transferred to PVDF membrane using a Trans‐Blot Turbo Transfer System (Bio‐Rad), then blocked with 5% milk and 1% BSA in buffer consisting of 150 mmol/L NaCl, 20 mmol/L Tris–HCl, pH 7.5, and 0.1% (v/w) Tween for 1 h. The blot was probed with either PDX1 (1:500, #2437, Cell Signaling Technology, Danvers, MA, USA), SYT11 (1:500; #ab204589, Abcam, UK), SYT13 (1:500; #ab154695, Abcam, UK), or Cyclophilin B (1:2000; # ab16045 Abcam, UK) antibody and incubated overnight at 4°C. Horseradish peroxidase‐conjugated goat anti‐rabbit IgG, HRP‐linked antibody (1:1000; #170‐6515; Bio‐Rad, Hercules, CA, USA) was used to detect the primary antibodies. Clarity Western ECL Substrate was used for visualization of proteins with a ChemiDoc XRS+ System (Bio‐Rad). The signal intensity of each protein band was measured using an Image Lab software (ver. 6.0.1; Bio‐Rad) and normalized to that of the total protein bands in the lane or Cyclophilin B expression.

### 4.8 Electrophysiology

Patch pipettes were pulled from borosilicate glass capillaries, coated with sticky wax (Kemdent, UK), and fire‐polished. The pipette resistance was 3–6 MΩ when they were filled with the pipette solution. To measure ion channel currents and exocytosis (as changes in membrane capacitance) whole‐cell patch‐clamp experiments on single cells were performed as previously described^34^ with a pipettes solution containing (mM): 125 Cs‐Glutamate, 10 NaCl, 10 CsCl, 1 MgCl2, 0.05 EGTA, 3 Mg‐ATP, 5 HEPES (pH 7.15 using CsOH) and 0.1 cAMP and an extracellular solution with (mM): 118 NaCl, 20 TEA‐Cl, 5.6 KCl, 2.6 CaCl2, 1.2 MgCl2, 5 glucose, and 5 HEPES (pH 7.4 using NaOH). The recordings were performed using patch master software (version 2‐73) and EPC‐10 amplifier (Heka Elektronik, Lambrecht, Germany). Exocytosis was evoked by a train of ten 500‐ms depolarisation from −70 mV to 0 mV applied at 1 Hz. Voltage‐dependent currents were investigated using an IV‐protocol in which the membrane was depolarized from −70 mV to voltages between −40 mV to +40 mV during 50 ms. Charge (Q) was measured ~2 ms after the onset of the pulse to exclude the rapidly inactivating Na^+^‐current and is, therefore, representative of the Ca^2+^‐influx.

### 4.9 Data analysis

Processed RNA‐sequencing data as previously described in Ref. [15] were obtained from publicly available repositories (GSE50398, GSE108072). Expression is shown as reads per kilobase million (RPKM) gene expression values. Spearman correlations between *SYT* and *PDX‐1* gene expression or phenotypic traits were performed using their RPKM gene expression values.

Data are given as mean ± SEM unless otherwise indicated. Statistical significance was evaluated using two‐tailed Student's *t*‐test. Multiple testing in qPCR experiments was corrected with the Holm‐Sidak method or two‐stage step‐up (Benjamini, Krieger, and Yekutieli) except for Figure 8A where an ANOVA was used. Multiple comparisons in insulin secretion experiments were evaluated using two‐way ANOVA. All statistical calculations were performed using GraphPad Prism version 8 and 9.

## CONFLICT OF INTEREST

The authors declare no conflict of interest.

## Data Availability

The data that supports the findings of this study are available from the corresponding authors upon reasonable request or openly available at Gene Expression Omnibus (accession no. GSE50398 and GSE108072).
